# Potential role of irisin-mediated oxidative stress defense in systemic diseases

**DOI:** 10.3389/fcell.2026.1996659

**Published:** 2026-10-13

**Authors:** Xue Xiao, Liying Wu, Xiaoming Zhu, Jie Zhou

**Affiliations:** 1 Department of Cardiology, The Affiliated Hospital of Changchun University of Chinese Medicine, Changchun, Jilin, China; 2 Department of Respiratory Medicine, The Affiliated Hospital of Changchun University of Chinese Medicine, Changchun, Jilin, China

**Keywords:** antioxidant, irisin, mitochondria, oxidative stress, reactive oxygen species

## Abstract

Oxidative stress arises when excessive reactive oxygen species (ROS) overwhelm endogenous antioxidant defenses, leading to deleterious modifications of lipids, proteins, and DNA. Persistent oxidative stress is implicated in the pathogenesis of numerous chronic disorders, including cardiovascular diseases, neurodegenerative conditions, type 2 diabetes mellitus, and ischaemia–reperfusion (I/R) injury across multiple organs. Irisin, a myokine/adipokine proteolytically cleaved from fibronectin type III domain-containing protein 5 (FNDC5), was first characterized in 2012. Preclinical studies have increasingly demonstrated that irisin possesses potent antioxidant properties and exerts cytoprotective effects in diverse tissues. These antioxidant actions are mediated through multiple molecular mechanisms: activation of the nuclear factor erythroid 2-related factor 2 (Nrf2) pathway with subsequent upregulation of cytoprotective enzymes such as haem oxygenase-1 (HO-1); sirtuin 3 (SIRT3)-dependent deacetylation and activation of mitochondrial superoxide dismutase 2 (SOD2); inhibition of pro-oxidant enzymes including NADPH oxidase (NOX); and modulation of mitochondrial fusion-fission dynamics to preserve mitochondrial homeostasis. Functionally, irisin initiates intracellular signalling cascades, including AMP-activated protein kinase (AMPK), PI3K/Akt, and MAPK pathways, primarily through binding to cell-surface integrin αV receptors. This review systematically summarizes the molecular mechanisms underlying irisin-mediated antioxidation, delineates its organ-protective roles in cardiovascular, neurological, metabolic, hepatic, renal, pulmonary, gastrointestinal, skeletal, reproductive systems, as well as in sepsis and ageing, and discusses its translational potential as a clinical biomarker and therapeutic target for oxidative-stress-associated diseases.

## 1 Introduction

Reactive oxygen species are continuously generated as physiological by-products of aerobic metabolism. Under basal conditions, endogenous antioxidant machinery maintains redox homeostasis. However, when ROS production exceeds the capacity of antioxidant defenses—whether due to increased generation or impaired scavenging—accumulated free radicals trigger lipid peroxidation, protein denaturation, DNA strand breakage, and ultimately programmed cell death (Zorov et al., 2014). Oxidative stress constitutes a central pathogenic mechanism underlying a wide spectrum of life-threatening illnesses, including atherosclerosis (Jiang et al., 2023), myocardial infarction (Zheng et al., 2022), ischaemic stroke (Zhang Y. et al., 2023), Alzheimer’s disease (Ionescu-Tuck et al., 2021), Parkinson’s disease (He et al., 2024), type 2 diabetes (De Marañon et al., 2020), non-alcoholic fatty liver disease (NAFLD) (Lv et al., 2024), acute kidney injury (AKI) (Zhao et al., 2021), and acute lung injury (ALI) (Li F. et al., 2024). Despite substantial advances in redox biology, clinically effective antioxidant-targeted therapies remain limited, highlighting an urgent need to identify novel endogenous antioxidant molecules with translational potential.

Discovered by Boström et al., in 2012, irisin is a cleavage product of the transmembrane precursor protein FNDC5, secreted into circulation primarily by skeletal muscle in response to physical exercise (Boström et al., 2012). Initially characterized for its role in driving white-to-brown adipose tissue browning, irisin has since been shown to exert pleiotropic biological activities extending far beyond metabolic regulation, encompassing anti-inflammatory, neuroprotective, cardioprotective, and osteoprotective effects (Boström et al., 2012; Lourenco et al., 2019; Zhang Z. et al., 2025; Tao et al., 2024). These actions are mediated through an intricate molecular network involving receptor-dependent signal transduction, transcriptional regulation of antioxidant genes, mitochondrial quality control, and suppression of pro-oxidant enzyme activity. The present review comprehensively examines the molecular mechanisms of irisin-driven antioxidation, summarizes its tissue-specific protective functions in oxidative-stress-related pathologies, and evaluates its feasibility as a diagnostic biomarker and pharmacological candidate for future clinical translation.

## 2 Biosynthesis, structural characteristics, and signal-transduction mechanisms of irisin

### 2.1 Biosynthesis and structural features

The full-length FNDC5 protein comprises 209 amino acids, consisting of an N-terminal 29-amino-acid signal peptide, a 94-amino-acid fibronectin type-III domain, a linker peptide, a transmembrane helix, and an intracellular C-terminal tail. Irisin corresponds to the 112-amino-acid extracellular fragment proteolytically released from FNDC5 (Boström et al., 2012). Crystallographic analyses have demonstrated that mature irisin exists as a stable homodimer, in which two subunits are tightly associated via β-sheet interactions, a unique structural feature among FNIII-domain-containing proteins. Asparagine-linked glycosylation occurs at residues Asn-7 and Asn-52, contributing to molecular heterogeneity; glycosylated irisin isoforms frequently display enhanced biological activity (Schumacher et al., 2013; Zhang et al., 2014).

Exercise-induced upregulation of peroxisome proliferator-activated receptor-γ coactivator-1α (PGC-1α) in skeletal muscle transcriptionally activates FNDC5 expression and stimulates irisin secretion (Boström et al., 2012). However, irisin is not exclusively a myokine, as it is also expressed in adipose tissue, myocardium, brain, liver, and kidney, indicating that locally produced irisin may exert autocrine and paracrine regulatory functions within these peripheral organs (Aydin et al., 2014).

### 2.2 Receptor-mediated downstream signalling cascades

Upon receptor engagement, multiple intracellular signalling pathways are activated. Kim et al. demonstrated that irisin binds to integrin αVβ5 on osteocytes and adipocytes, with this interaction essential for irisin-induced signaling (Kim et al., 2018), and subsequent studies confirmed that integrin αVβ1 and αVβ5 serve as primary receptors for irisin across diverse cell types (Liu S. et al., 2022). Upon binding, irisin activates several intracellular signaling pathways: the AMPK pathway, wherein irisin-integrin interaction triggers AMPK phosphorylation at Thr172, leading to downstream activation of PGC-1α, mitochondrial biogenesis, and inhibition of mTOR-mediated inflammatory responses (Liu S. et al., 2022; So and Leung, 2016), with AMPK activation also promoting Nrf2 nuclear translocation and antioxidant gene expression (Wen et al., 2025); the PI3K/Akt pathway, through which irisin stimulates PI3K/Akt signaling to enhance cell survival, inhibit apoptosis, and modulate glucose metabolism (Wu Y. et al., 2023), and in endothelial cells this pathway reduces oxidative/nitrative stress and improves vascular function (Zhu et al., 2016); the MAPK pathways, wherein both p38 MAPK and ERK1/2 are activated by irisin to mediate its effects on adipocyte browning, osteoblast proliferation, and anti-inflammatory responses (Zhang et al., 2014; Qiao et al., 2016); the STAT3 pathway, through which irisin promotes STAT3 phosphorylation and nuclear translocation to contribute to its antioxidant effects in pancreatic β-cells and other tissues (Liu C. et al., 2022); and the FAK pathway, wherein focal adhesion kinase activation downstream of integrin engagement mediates irisin’s effects on cell migration and tissue repair (Legar et al., 2025) (Figure 1).

**FIGURE 1:**
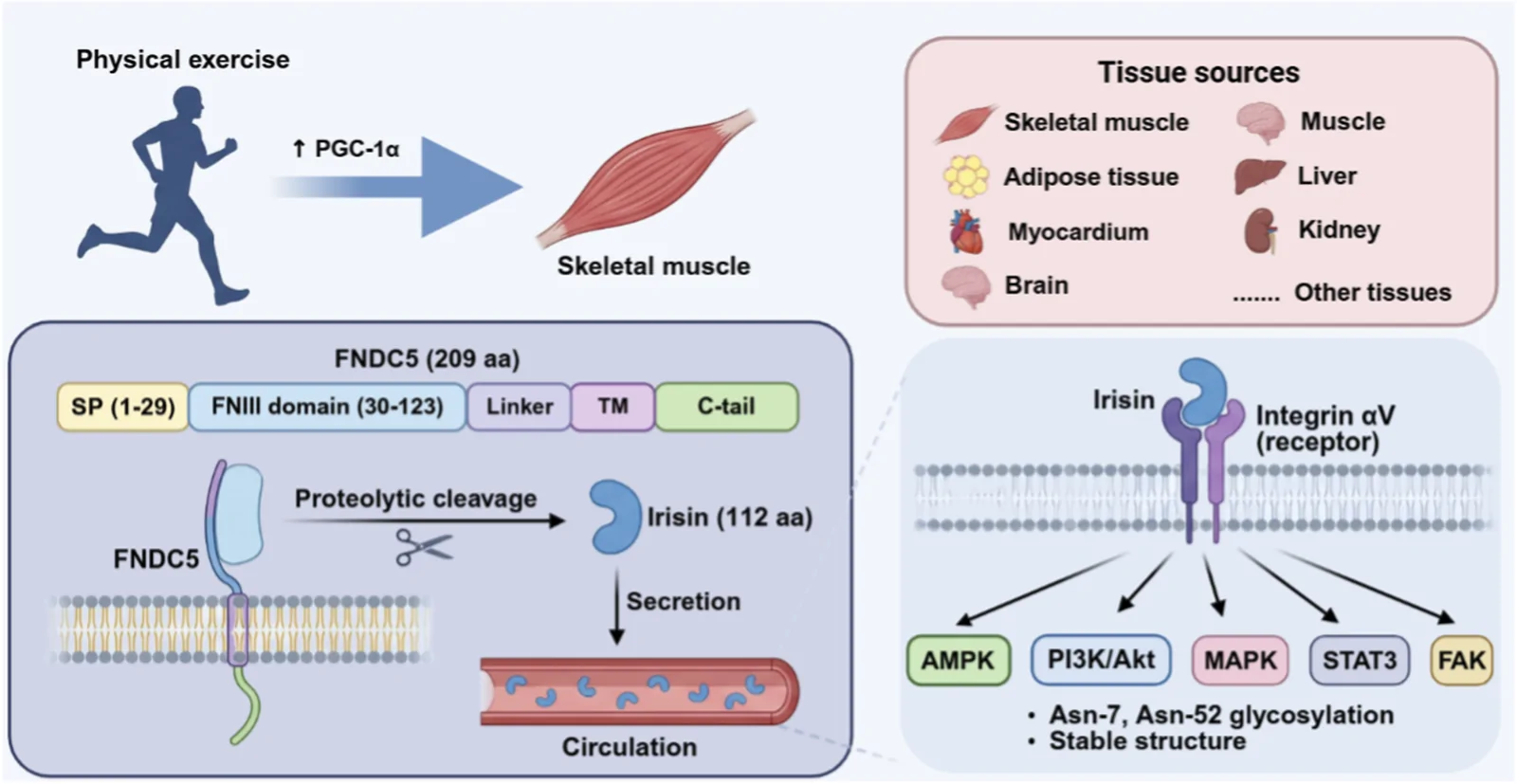
Biosynthesis, tissue sources, and receptor-mediated signalling of irisin. Exercise induces PGC-1α upregulation in skeletal muscle, promoting FNDC5 expression and irisin secretion. Full-length FNDC5 (209 aa) is proteolytically cleaved to release mature irisin (112 aa) into the circulation. Besides skeletal muscle, irisin is also produced by adipose tissue, myocardium, brain, liver, and kidney, acting in an autocrine/paracrine manner. Irisin binds mainly to cell-surface integrin αV receptors, activating downstream pathways including AMPK, PI3K/Akt, MAPK, STAT3, and FAK.

## 3 Molecular mechanisms underlying irisin-mediated antioxidant effects

### 3.1 Activation of the canonical Nrf2/ARE antioxidant signalling axis

The Nrf2/ARE pathway serves as the master transcriptional regulator of cellular antioxidant defence. Under physiological conditions, Nrf2 is sequestered in the cytoplasm by Kelch-like ECH-associated protein 1 (Keap1) and undergoes rapid ubiquitin-dependent proteasomal degradation (Ma, 2013). Upon oxidative insult, Nrf2 dissociates from Keap1, translocates into the nucleus, and binds to antioxidant-response elements (AREs) to initiate transcription of cytoprotective genes, including *HO-1*, *NQO1*, *GPX4*, and *catalase* (Mazur-Bialy et al., 2021).

Multiple *in vitro* and *in vivo* studies have confirmed that irisin robustly activates Nrf2 signalling. Mazur-Bialy et al. demonstrated that irisin treatment elevates Nrf2 and HO-1 protein levels in lipopolysaccharide-stimulated macrophages, promoting ROS scavenging and attenuating inflammatory injury (Mazur-Bialy et al., 2021). In hepatic and cerebral I/R injury models, irisin pre-treatment upregulates nuclear Nrf2 abundance, reduces malondialdehyde (MDA) concentrations, and restores superoxide dismutase (SOD) and glutathione (GSH) activity in a dose-dependent manner (Bi et al., 2019). Pharmacological inhibition of AMPK markedly attenuates irisin-facilitated Nrf2 nuclear translocation, suggesting that AMPK acts as an upstream mediator of this antioxidant cascade (Cheng et al., 2026). Recently, a novel integrin–JAK2–STAT6–Nrf2 signalling axis was identified: irisin–integrin αVβ5 binding triggers JAK2 phosphorylation, which subsequently activates STAT6 to amplify Nrf2-dependent antioxidant gene transcription (Tu et al., 2023).

### 3.2 SIRT3-dependent enhancement of mitochondrial antioxidant capacity via SOD2 deacetylation

Mitochondria constitute the predominant intracellular source of ROS, and mitochondrial manganese-dependent superoxide dismutase (SOD2, MnSOD) is the primary enzyme responsible for detoxifying mitochondrial superoxide anions (Palma et al., 2024). SIRT3, an NAD^+^-dependent mitochondrial deacetylase, removes inhibitory acetyl groups from SOD2, thereby augmenting SOD2 enzymatic activity and mitochondrial ROS clearance (Xu et al., 2020). In cerebral I/R injury, irisin restores SIRT3 expression and activity in a dose-dependent manner, reversing excessive SOD2 acetylation and reducing mitochondrial ROS generation. Blockade of SIRT3 with the selective inhibitor 3-TYP abolishes irisin-mediated neuroprotection, confirming that SIRT3 is an essential downstream effector of irisin-mediated mitochondrial antioxidation (Cong et al., 2025). In diabetic adipose-derived stem cells, irisin enhances osteogenic differentiation via SIRT3-regulated mitochondrial respiration and modulation of SOD2 acetylation status (Li et al., 2022). This SIRT3-SOD2 antioxidant cascade also underpins irisin-induced neuroprotection against traumatic brain injury, where irisin preserves mitochondrial membrane potential and suppresses mitochondrial superoxide overproduction (Ge et al., 2024). Collectively, SIRT3 functions as a core hub linking irisin signalling to mitochondrial antioxidant defence.

### 3.3 Modulation of the thioredoxin antioxidant system

The thioredoxin (Trx) system, consisting of Trx, thioredoxin reductase (TrxR), and peroxiredoxin (Prx), represents another vital intracellular reducing system for ROS elimination (Jia et al., 2019). Thioredoxin-interacting protein (Txnip) is an endogenous negative regulator of Trx, and Txnip overexpression exacerbates oxidative cellular injury (Xu et al., 2013). Zhao et al. identified a previously uncharacterized antioxidant mechanism in pancreatic β-cells exposed to lipotoxic oxidative stress. Palmitic acid treatment of INS-1 β-cells increases Txnip abundance and suppresses mitochondrial Trx2 activity, whereas irisin intervention reverses Txnip upregulation and restores functional Trx2. Mechanistically, irisin promotes STAT3 phosphorylation and nuclear translocation; siRNA-mediated STAT3 knockdown abolishes irisin-driven Trx2 activation. *In vivo* experiments in high-fat-diet-fed mice further validated that recombinant irisin administration reduces pancreatic MDA levels, enhances glutathione peroxidase (GPx) activity, and preserves β-cell function via the STAT3–Trx2 signalling cascade. This Txnip/STAT3/Trx2 axis constitutes a tissue-specific antioxidant pathway for irisin within metabolically active organs (Liu C. et al., 2022).

### 3.4 Maintenance of mitochondrial dynamics and mitochondrial quality-control programmes

Mitochondrial dysfunction and excessive ROS production form a vicious pathogenic cycle. Irisin promotes mitochondrial fusion and inhibits pathological mitochondrial fission, thereby preserving mitochondrial structural integrity and attenuating ROS overgeneration. In H_2_O_2_-challenged cardiomyocytes, irisin stabilizes mitochondrial membrane potential, lowers intracellular ROS concentrations, and restores energy metabolism, accompanied by upregulation of fusion-related proteins OPA1 and MFN2 and downregulation of the fission mediator DRP1. Silencing of PGC-1α partially diminishes these protective effects (Wang et al., 2024). During cerebral I/R injury, irisin suppresses DRP1-mediated mitochondrial fission and restores Mfn2 and Opa1 expression to reduce neuronal apoptosis, with upstream regulation via the PI3K/Akt/mTOR pathway (Liu et al., 2023). Meanwhile, irisin promotes mitochondrial biogenesis by upregulating the PGC-1α/TFAM axis, increasing mitochondrial content. These biogenic effects are mediated through integrin receptor–dependent Akt/ERK1/2 signaling and are partially dependent on UCP-2-related targets (Bi et al., 2019; Tu et al., 2021; Zhang X. et al., 2023). In parallel, irisin activates PINK1/Parkin-dependent mitophagy to clear damaged, ROS-producing mitochondria (Li H. et al., 2021; Ding and Zhang, 2023). These coordinated processes sustain mitochondrial quality and mitigate oxidative stress.

### 3.5 Repression of pro-oxidant enzyme activity

Beyond reinforcing endogenous antioxidant defences, irisin directly suppresses enzymes responsible for ROS biosynthesis. NADPH oxidase (NOX) is a major source of superoxide anions in vascular and inflammatory cells (Chang et al., 2017; Szeto et al., 2008). In diabetic cardiomyopathy, irisin inhibits NOX2 (Lin et al., 2021) and reverses the downregulation of GPx and SOD1 in RAW 264.7 mouse macrophages (Mazur-Bialy et al., 2021). In high-glucose–high-fat-treated endothelial cells, irisin inhibits the PKC-β/NOX axis to reduce ROS generation (Zhu et al., 2015). Additionally, irisin modulates nitric oxide (NO) homeostasis. Under post-stroke, irisin upregulates endothelial nitric-oxide synthase (eNOS) expression to increase bioavailable NO, while reducing cytotoxic peroxynitrite formation, thereby improving endothelial function and alleviating oxidative–nitrosative stress (Guo et al., 2025) (Table 1).

**TABLE 1:** Summary of molecular mechanisms underlying irisin-mediated antioxidant effects.

Mechanism	Key pathways	Major outcomes	References
Nrf2/ARE transcriptional activation	Keap1, Nrf2, HO-1, NQO1, GPX4, catalase; AMPK; integrin-JAK2-STAT6	Upregulates phase II detoxifying enzymes; reduces ROS and MDA; increases GSH and SOD; promotes Nrf2 nuclear translocatio	(Ma, 2013; Mazur-Bialy et al., 2021; Bi et al., 2019; Cheng et al., 2026; Tu et al., 2023)
SIRT3-dependent SOD2 deacetylation	SIRT3, SOD2 (MnSOD)	Enhances mitochondrial superoxide scavenging; preserves mitochondrial membrane potential; reduces mitochondrial ROS	(Palma et al., 2024; Xu et al., 2020; Cong et al., 2025; Li et al., 2022; Ge et al., 2024)
Thioredoxin system modulation	Trx2, Txnip, STAT3	Restores Trx2 activity; lowers Txnip; reduces lipotoxic oxidative stress in pancreatic β-cells; enhances GPx activity	(Liu et al., 2022b; Jia et al., 2019; Xu et al., 2013)
Mitochondrial dynamics and quality control	DRP1, MFN2, OPA1, PGC-1α, TFAM, PINK1/Parkin, UCP2	Promotes mitochondrial fusion; inhibits pathological fission; stimulates mitophagy and mitochondrial biogenesis; reduces mitochondrial ROS	(Bi et al., 2019; Wang et al., 2024; Liu et al., 2023; Tu et al., 2021; Zhang et al., 2023b; Li et al., 2021a; Ding and Zhang, 2023)
Repression of pro-oxidant enzymes	NOX2, NOX4, PKC-β/NOX, xanthine oxidase; eNOS	Suppresses superoxide production; decreases peroxynitrite formation; restores NO bioavailability; improves endothelial function	(Chang et al., 2017; Szeto et al., 2008; Lin et al., 2021; Zhu et al., 2015; Guo et al., 2025)

## 4 Protective role of irisin in systemic diseases

Given that oxidative stress represents a shared pathogenic driver of multiorgan damage, irisin functions as a versatile endogenous cytoprotector, deploying tissue-specific antioxidant strategies across distinct organ systems.

### 4.1 Cardiovascular system

#### 4.1.1 Myocardial ischaemia–reperfusion injury

Myocardial I/R injury is characterized by reperfusion-evoked ROS overproduction and mitochondrial dysfunction that drives cardiomyocyte death (Zhao et al., 2018). The underlying mechanisms involve electron transport chain dysfunction, xanthine oxidase activation, and neutrophil infiltration, all of which amplify ROS generation. The cardioprotective antioxidant effects of irisin have been well documented in preclinical I/R models. Lu et al. demonstrated that 100 ng/mL irisin improves left-ventricular ejection fraction and lowers serum troponin levels in a dose-dependent manner. Mechanistically, irisin mitigates I/R-provoked oxidative stress, stabilizes mitochondrial membrane potential, and attenuates mitochondrial superoxide bursts. Notably, irisin selectively binds SOD2 (but not SOD1) and facilitates correct mitochondrial localization of SOD2 under hypoxia–reoxygenation conditions (Wang et al., 2018). Wang et al. further reported that irisin exerts anti-apoptotic effects by upregulating SOD1 and inducing p38 phosphorylation (Wang et al., 2017). Irisin also enhances PINK1/Parkin-dependent mitophagy to eliminate dysfunctional mitochondria, and inhibits ER stress, ROS production through upregulation of Mitochondrial ubiquitin ligase, thereby conferring multimodal myocardial protection (Li H. et al., 2021; Lu et al., 2020). In addition, irisin attenuates endoplasmic reticulum stress and reduces the expression of pro-apoptotic Bax and cleaved caspase-3, limiting infarct size and preserving cardiac function (Ren et al., 2022). Irisin also attenuates sepsis-induced cardiac dysfunction by inhibiting inflammation-induced pyroptosis through a mitochondrial ubiquitin ligase (MITOL)-dependent mechanism (Xiong et al., 2022).

#### 4.1.2 Atherosclerosis

Atherosclerosis is driven by chronic vascular inflammation and oxidative stress, with endothelial dysfunction—characterized by reduced NO bioavailability and excess ROS—constituting its initiating event (Baynard et al., 2023). Oxidized low-density lipoprotein (ox-LDL) uptake by macrophages promotes foam cell formation and further enhances ROS production via NOX activation, establishing a pro-atherogenic milieu. Zhu et al. reported that irisin ameliorates type 2-diabetes-associated endothelial dysfunction largely by blunting oxidative-nitrative stress via repression of the PKC-β/NADPH oxidase and NF-κB/iNOS axes, achieving dual-target endothelial protection (Zhu et al., 2015). In high-cholesterol-fed ApoE-knockout mice, irisin reduces aortic atherosclerotic plaque burden by blocking ox-LDL-triggered ROS–p38-MAPK–NF-κB signalling and promoting endothelial repair through upregulation of microRNA-126–5p (Zhang et al., 2016a; Zhang et al., 2016b). Moreover, irisin inhibits vascular smooth muscle cell proliferation and migration—key processes in neointima formation—by attenuating oxidative stress and modulating matrix metalloproteinase activity (Wang J. et al., 2026). Clinically, in Behçet’s disease patients, decreased serum irisin levels are associated with subclinical atherosclerosis, suggesting that irisin may serve as a potential biomarker for early atherosclerotic vascular involvement in autoimmune disorders (Ismail et al., 2023).

#### 4.1.3 Diabetic cardiomyopathy

Diabetic cardiomyopathy is defined as a progressive cardiac dysfunction characterized by mitochondrial oxidative stress, defective autophagy, and interstitial fibrosis, occurring in the absence of coronary artery disease or hypertension. Chronic hyperglycaemia enhances the polyol and hexosamine pathways, generating advanced glycation end-products (AGEs) that activate receptor for AGE (RAGE)-dependent NADPH oxidase, perpetuating myocardial ROS production. Lin et al. demonstrated that FNDC5/irisin activates the integrin αVβ5–Akt signalling pathway, attenuates oxidative–nitrosative stress, stabilizes mitochondrial membrane potential, and improves myocardial contractility (Lin et al., 2021). Li et al. further revealed that irisin accelerates the clearance of damaged mitochondria and oxidized proteins through AMPK/mTOR-dependent autophagy, diminishing the primary source of intracellular ROS (Deng et al., 2020). Additionally, irisin reduces cardiac fibrosis by inhibiting TGF-β1/Smad signalling and suppressing fibroblast-to-myofibroblast transformation, thereby ameliorating diastolic dysfunction in diabetic hearts (Chen et al., 2019).

### 4.2 Nervous system

#### 4.2.1 Cerebral ischaemia–reperfusion injury

Cerebral I/R injury provokes excessive ROS generation and mitochondrial impairment, which promote neuronal damage. Irisin exhibits potent neuroprotective activity in experimental stroke models, including middle cerebral artery occlusion (MCAO) mice and oxygen-glucose deprivation/reoxygenation (OGD/R) neuronal cell models. Cong et al. showed that systemic irisin administration (50–100 μg/kg) restores SIRT3 expression and activity, decreases SOD2 acetylation, and suppresses mitochondrial ROS accumulation in a dose-dependent manner. Neuroprotection was completely abolished by SIRT3 inhibition, confirming its indispensable role. Concurrently, irisin activates Nrf2 signalling, elevates GSH levels, and reduces MDA concentrations (Cong et al., 2025). *In vitro*, irisin recapitulated these protective effects against oxygen–glucose deprivation-induced injury in PC12 neurons via modulation of the ROS-NLRP3 inflammatory signaling pathway (Peng et al., 2017). Irisin also modulates mitochondrial dynamics via PI3K/Akt/mTOR, restraining DRP1-mediated fission and upregulating Mfn2/Opa1-driven fusion to inhibit neuronal apoptosis (Liu et al., 2023). Additionally, exercise-stimulated circulating irisin may promote post-stroke cognitive recovery by regulating the anti-ageing protein klotho, which suppresses oxidative damage and enhances synaptic plasticity (Jin et al., 2021).

#### 4.2.2 Parkinson’s disease

Parkinson’s disease (PD) features selective degeneration of nigral dopaminergic neurons, with mitochondrial complex I deficiency and oxidative stress as core pathological hallmarks. The accumulation of α-synuclein oligomers further impairs mitochondrial function and triggers endoplasmic reticulum stress, creating a feedforward loop of ROS production. In α-synuclein pre-formed fibril models, irisin inhibits NLRP3 inflammasome activation, microglial overactivation, and oxidative injury (Zhu et al., 2025). In the MPTP/MPP^+^ PD model, irisin improves mitochondrial function, boosts ATP synthesis, and reduces ROS burden to safeguard dopaminergic neurons via the sirtuin 1 (SIRT1)/HIF-1α/PGC-1α signalling pathway (Cai et al., 2025).

#### 4.2.3 Traumatic brain injury

Secondary injury following traumatic brain injury (TBI) involves oxidative stress, neuroinflammation, and blood–brain barrier (BBB) disruption, with mitochondrial ROS overproduction serving as a central pathogenic driver. Serum irisin levels were found to significantly correlate with neurological outcomes in TBI patients. Mechanistic studies in TBI mice have revealed that FNDC5/irisin protects against TBI by promoting SIRT3-dependent mitochondrial quality control and oxidative stress inhibition (Ge et al., 2024). Complementary evidence from Guo et al. demonstrated that irisin reduces neuronal mitochondrial ROS production by upregulating uncoupling protein 2 (UCP2) and preserves BBB integrity (Guo et al., 2021). Beyond direct ROS suppression, irisin improves neurological function and reduces neuronal apoptosis by attenuating oxidative stress through ROS inhibition and SYK suppression, while concomitantly activating the AMPK/MerTK-mediated autophagy pathway to inhibit pyroptosis and neuroinflammation (Zhang K. et al., 2025). Collectively, these findings establish oxidative stress mitigation as a central mechanism through which irisin exerts multimodal neuroprotection against TBI.

#### 4.2.4 Prion disease

A recent study by Wen et al. reported that irisin rescues prion-peptide-triggered neuronal apoptosis by targeting UCP2 and activating the AMPK–Nrf2 cascade to restore mitochondrial homeostasis. This finding extends the neuroprotective spectrum of irisin to protein-misfolding-mediated neurodegenerative disorders, underscoring its broad applicability in conditions where oxidative stress and mitochondrial failure are central to neuronal death (Li et al., 2023).

#### 4.2.5 Sepsis-associated encephalopathy

Sepsis-associated encephalopathy (SAE) involves neuroinflammation, oxidative stress, and neuronal ferroptosis. Irisin protects against SAE by suppressing lipid ROS and increasing mitochondrial membrane potential via activation of the Nrf2/GPX4 signalling axis, thereby alleviating cognitive impairment, neurological severity scores, and microglial activation. Notably, irisin-mediated anti-ferroptosis was abolished by siRNA-Nrf2 or in Nrf2^−/−^ mice, confirming Nrf2 as an essential mediator (Wang J. et al., 2022).

### 4.3 Metabolic system

Pancreatic β-cells are inherently vulnerable to oxidative damage owing to low endogenous antioxidant enzyme reserves and high metabolic activity. This oxidative burden impairs insulin gene transcription, reduces glucose-stimulated insulin secretion, and promotes β-cell apoptosis. Zhao et al. demonstrated that Irisin protects pancreatic β-cells against palmitic acid-elicited lipotoxic damage both *in vitro* and *in vivo* by suppressing Txnip, activating the Stat3-Trx2 axis to mitigate oxidative stress, thereby restraining β-cell apoptosis and preserving insulin-secretory function (Liu C. et al., 2022). Additional research has shown that irisin inhibits NOD-like receptor protein 3 (NLRP3)/gasdermin D (GSDMD)-mediated pyroptosis and activates the Nrf2–Trx/Txnip axis to defend β-cells (Li et al., 2023). Clinically, circulating irisin concentrations are significantly reduced in patients with type 2 diabetes and correlate negatively with blood glucose, serum lipids, and visceral fat mass, suggesting a potential role in disease monitoring (Liu et al., 2013).

### 4.4 Hepatic system

#### 4.4.1 Non-alcoholic fatty liver disease

NAFLD represents the hepatic manifestation of metabolic syndrome, and oxidative stress drives disease progression from simple steatosis to steatohepatitis and liver fibrosis. The liver itself constitutes a local source of irisin, which exerts autocrine hepatoprotective effects (Canivet et al., 2020). Serum irisin levels are reduced in NAFLD patients and negatively correlated with intrahepatic triglyceride content (Li et al., 2022; Mertoğlu et al., 2025; Daneshi-Maskooni et al., 2019; Zhang et al., 2013). Circulating irisin concentrations independently correlate with portal inflammation and hepatic redox status in NAFLD cohorts, suggesting dual potential as a biomarker and therapeutic target (Polyzos et al., 2014). Hepatic FNDC5/irisin upregulation mitigates high-fat-diet-induced hepatic steatosis and hepatocyte apoptosis by activating AMPKα and repressing the lipogenic transcription factor SREBP-1c (Yu et al., 2022). exercise-induced irisin inhibits inflammation via competitive binding with MD2 to improve NAFLD (Zhu et al., 2021) and contribute to Antioxidant Effects of Silymarin in Dexamethasone- Induced NAFLD (Ghasemi et al., 2025).

#### 4.4.2 Hepatic ischaemia–reperfusion injury

Hepatic ischaemia–reperfusion (I/R) injury causes massive ROS release and mitochondrial dysfunction, leading to hepatocyte demise. Bi et al. reported that irisin reduces pathological mitochondrial fission, enhances mitochondrial fusion and biogenesis, and attenuates oxidative stress in an integrin-αV-dependent manner, via downregulation of DRP1 and upregulation of MFN2 and PGC-1α. Irisin also inhibits pro-apoptotic JNK signalling, upregulates telomerase activity, and increases GPX4 expression to counteract lipid peroxidation and ferroptosis, a recently recognized iron-dependent cell death pathway (Bi et al., 2019).

#### 4.4.3 Liver fibrosis

Hepatic fibrosis results from chronic injury that drives HSC activation, transforming quiescent vitamin-A-storing cells into myofibroblast-like cells that secrete excessive extracellular matrix. Recombinant irisin suppresses TGF-β1-stimulated HSC activation (Dong et al., 2020), in bile-duct-ligation-induced hepatic fibrosis mouse models, irisin alleviates HSC activation and fibrogenesis by blocking TGF-β1/Smad-driven ROS generation, restoring mitochondrial fusion–fission balance, stabilizing mitochondrial membrane potential, and improving ATP production (Lai et al., 2024).

#### 4.4.4 Sepsis-associated liver injury

In sepsis-related hepatic damage, irisin suppresses ferroptosis and restores mitochondrial function. Serum irisin levels are decreased and negatively correlated with APACHE II scores APACHE II, SOFA scores and 28-day mortality risk in septic patients. In caecal ligation and puncture (CLP)-induced septic mice, exogenous irisin suppressed ferroptosis, inhibited inflammatory responses, decreased ROS production, restored abnormal mitochondrial morphology, and increased mtDNA copy number and ATP content; these protective effects were reversed by GPX4 inhibition, while blocking the irisin receptor with RGD peptide or Echistatin decreased irisin-induced GPX4 expression (Wei et al., 2020). Irisin also alleviates LPS-induced liver injury and inflammation through inhibition of NLRP3 inflammasome activation and NF-κB signalling (Li Q. et al., 2021).

### 4.5 Renal system: acute kidney injury

Renal ischaemia-reperfusion (I/R) injury induces robust ROS burst and mitochondrial damage, resulting in tubular epithelial cell injury. Qin et al. demonstrated that irisin pre-treatment protects against I/R-induced AKI via upregulating mitochondrial UCP2 through AMPK signalling, suppressing renal ROS generation, lowering pro-inflammatory cytokine levels, reducing MDA, and restoring SOD activity (Zhang et al., 2020). Consistently, Irisin treatment alleviated renal injury, downregulated inflammatory response, improved mitochondrial function, and reduced ER stress and oxidative stress after renal I/R, which were associated with upregulation of GPX4 (Zhang et al., 2021). In addition, irisin alleviates AKI by activating PGC-1α–mediated autophagy, thereby mitigating inflammation, lipid accumulation, oxidative stress, and cellular damage (Han et al., 2026). In sepsis-associated AKI, post-treatment with irisin by reducing ROS production, iron content, and MDA level and increasing GSH level, as well as the alteration of ferroptosis-related protein (GPX4 and ACSL4) expressions in renal tissue via the SIRT1/Nrf2 pathway, thereby preserving renal function and reducing histological damage (Qiongyue et al., 2022). Similarly, irisin attenuates renal injury and inflammation by activating the Nrf2/HO-1 pathway to inhibit oxidative stress and ferroptosis (Wang B. et al., 2026). FNDC5 overexpression further boosts p-AMPK/AMPK, Nrf2, and HO-1 protein concentrations, and irisin ameliorates LPS-induced SA-AKI by inhibiting ferroptosis through the AMPK/Nrf2 signalling pathway (Gui et al., 2024).

### 4.6 Respiratory system

#### 4.6.1 Acute lung injury

ALI/acute respiratory distress syndrome (ARDS) features alveolar epithelial damage, neutrophil infiltration, and widespread oxidative stress. Sepsis represents a major clinical trigger for acute lung injury/ARDS, where systemic inflammatory-oxidative cascades propagate pulmonary parenchymal damage. In sepsis-ALI mouse models, irisin ameliorates pulmonary histopathological damage, reduces pro-inflammatory cytokine secretion, reverses elevations in MDA and Fe^2+^, restores GSH reserves, and inhibits ferroptosis-related genes (*Acsl4*, *Ptgs2*) (Zhang F. et al., 2025). Han et al. reported that irisin restricts alveolar macrophage pyroptosis via suppression of the HSP90/NLRP3/caspase-1/GSDMD signalling cascade (Han et al., 2023), while Li et al. confirmed that irisin strengthens alveolar epithelial barrier function through AMPK/SIRT1 activation (Li et al., 2019).

#### 4.6.2 Lung ischaemia–reperfusion injury

Lung I/R injury triggers reperfusion-driven ROS overproduction, causing alveolar-capillary barrier disruption. Chen et al. revealed that irisin protects the alveolar–capillary barrier and reduces alveolar epithelial cell apoptosis by upregulating UCP2 to suppress mitochondrial ROS overproduction (Chen et al., 2017). Irisin postconditioning protects against lung ischaemia/reperfusion injury by attenuating oxidative stress, as evidenced by reduced ROS, MDA, and Fe^2+^ levels and restored GPX4/ACSL4 balance (Wang Y. et al., 2022).

#### 4.6.3 Chronic obstructive pulmonary disease

Patients with COPD display lower circulating irisin concentrations than healthy controls, and serum irisin increases following structured physical exercise training. A 2025 clinical study quantitatively confirmed that serum irisin levels in COPD patients were significantly lower than those in healthy controls. Furthermore, irisin expression levels were found to be an independent prognostic indicator for acute exacerbation in COPD patients (Ijiri et al., 2015). Decreased serum irisin levels are also related to emphysema in patients with COPD (Sugiyama et al., 2017), whereas treatment of COPD patients with long-acting muscarinic antagonist (LAMA) increases circulating irisin (Mandal et al., 2018). Mechanistically, in a mouse model of acute exacerbation of COPD, irisin directly bound to integrin αVβ5, maintained ER homeostasis via the PERK/ATF4/CHOP pathway, and activated AMPK (Bai et al., 2026). In cigarette-smoke-exposed COPD mouse models, exercise-induced endogenous irisin upregulation correlates with enhanced pulmonary Nrf2 and HO-1 expression, supporting the exercise–irisin–Nrf2 axis as a potential strategy for alleviating emphysematous lung damage. Irisin also reduces airway remodelling by inhibiting TGF-β1-induced epithelial–mesenchymal transition and fibroblast proliferation (Kubo et al., 2019).

#### 4.6.4 Asthma

Asthma is a chronic inflammatory airway disease characterized by airway hyperresponsiveness, inflammation, and subepithelial fibrosis, in which oxidative stress plays a pivotal role by driving airway remodeling and mitochondrial dysfunction in bronchial fibroblasts. Cheng et al. demonstrated that FNDC5/irisin levels are reduced in asthma-derived lung fibroblasts and in house dust mite (HDM)-induced asthmatic mice. Treatment with irisin decreased alpha-smooth muscle actin (α-SMA) expression, improved mitochondrial function, and suppressed TGF-β-induced fibrotic protein expression and Smad3 phosphorylation in human lung fibroblasts. Mechanistically, RNA sequencing confirmed that irisin enhanced antioxidant-related gene expression and activated the AMPK pathway, which in turn reduced TGF-β-induced Smad3 phosphorylation and fibronectin expression. Moreover, irisin induced Nrf2 phosphorylation, and Nrf2 knockdown diminished its protective effects on fibrosis markers (Cheng et al., 2026). *In vivo*, irisin treatment alleviated HDM-induced airway fibrosis, inflammation, and lung function impairments (Bai et al., 2026).

### 4.7 Gastrointestinal system

#### 4.7.1 Intestinal ischaemia–reperfusion injury

Intestinal I/R injury features reperfusion-associated ROS accumulation and mitochondrial dysfunction that disrupt epithelial integrity. Irisin confers protection against intestinal ischaemia/reperfusion (I/R) injury through multiple converging mechanisms. Du et al. demonstrated that irisin pretreatment ameliorated intestinal I/R and hypoxia/reoxygenation (H/R)-induced injury *in vivo* and *in vitro* by activating the Nrf2 pathway, as evidenced by reduced TNF-α, IL-1β, and IL-6 levels, decreased MDA and MPO, increased SOD and GSH-Px activities, downregulated Bax and cleaved caspase-3, and upregulated Bcl-2; Nrf2 siRNA partially abolished these protective effects (Du et al., 2019). Bi et al. further showed that irisin restored gut barrier function after intestinal I/R by binding to the integrin αVβ5 receptor and activating the AMPK–UCP2 pathway, thereby reducing oxidative stress, calcium influx, and endoplasmic reticulum stress, protecting mitochondrial function, and decreasing enterocyte apoptosis; inhibition of integrin αVβ5, AMPK, or UCP2 abolished the protective role of irisin (Bi et al., 2020). In addition, Fan et al. reported that irisin contributes to the hepatoprotective effect of dexmedetomidine during intestinal I/R, as irisin-neutralizing antibody partially abrogated the reduction in liver injury, NLRP3 inflammasome activation, oxidative stress, and apoptosis in rats subjected to intestinal I/R (Fan et al., 2019). Collectively, these findings indicate that irisin protects against intestinal I/R injury and associated remote organ damage through Nrf2 activation and integrin αVβ5–AMPK–UCP2 signalling, involving antioxidant, anti-inflammatory, anti-apoptotic, and barrier-preserving actions.

#### 4.7.2 Acute pancreatitis

Acute pancreatitis (AP) frequently triggers secondary intestinal injury that amplifies systemic inflammatory responses. The pathogenesis of AP involves premature trypsinogen activation, intrapancreatic ROS generation, and acinar cell necrosis. Irisin has emerged as a protective factor in pancreatic diseases through its antioxidative properties. Tang et al. demonstrated that serum irisin levels were significantly reduced in patients with acute pancreatitis (AP) and inversely correlated with disease severity; in a murine cerulein-induced AP model, irisin triggered an antioxidative stress programme via KEAP1 degradation, restoring redox balance by decreasing ROS accumulation and suppressing inflammatory mediator secretion in pancreatic tissues, whereas increased KEAP1 expression counteracted these antioxidative effects (Tang et al., 2025). Ren et al. further showed that irisin administration (50 or 250 μg/kg) significantly mitigated intestinal damage, reduced apoptosis, and attenuated oxidative and endoplasmic reticulum stress in mice with L-arginine-induced AP, while also downregulating serum TNF-α and IL-6 levels and alleviating injury in the pancreas, liver, and lung (Ren et al., 2019). In a murine model of chronic pancreatitis (CP) induced by repeated cerulein injections, Ren et al. reported that serum irisin levels were decreased, and irisin treatment significantly alleviated pancreatic injury and fibrosis in association with reduced oxidative and endoplasmic reticulum stress; in cultured pancreatic stellate cells, irisin directly inhibited TGF-β-induced α-SMA and collagen I expression, likely through downregulation of kindlin-2 and inhibition of the SMAD2/3 pathway (Ren et al., 2020). Collectively, these findings indicate that irisin protects against both acute and chronic pancreatitis through KEAP1-mediated antioxidative signalling, suppression of oxidative and ER stress, and inhibition of pancreatic stellate cell activation and fibrosis.

### 4.8 Skeletal system: osteoporosis and osteoprotection

Bone metabolism is highly sensitive to redox homeostasis; excess ROS promotes osteoblast apoptosis and inhibits osteogenic differentiation, while also stimulating osteoclastogenesis via receptor activator of nuclear factor-κB ligand (RANKL)-mediated NF-κB activation. Age-related and diabetic osteoporosis share oxidative stress as a common pathway. Irisin rescues advanced glycation end products-induced dysfunction of adipose-derived stem cells (ASCs) and restores their osteogenic capacity via a SIRT3-dependent pathway, which maintains mitochondrial homeostasis, controls oxidative stress, mitophagy and mitochondrial biogenesis (Li et al., 2022). *In vivo* animal studies by Colaianni et al. verified that irisin increases cortical bone mass. Clinical data indicate that circulating irisin concentrations are positively associated with bone mineral density and negatively correlated with fracture risk in post-menopausal women (Colaianni et al., 2015). Exercise-stimulated irisin enhances skeletal antioxidant defence via Nrf2 signalling and promotes osteocyte survival under mechanical loading (Hu et al., 2026). Beyond osteoporosis, irisin also exerts protective effects in osteoarthritis (OA). combined treatment with trolox, recombinant irisin (r-irisin), and resveratrol in primary osteoblasts derived from patients with osteoarthritis and osteoporosis significantly increased cell viability, reduced ROS levels and senescence-associated β-galactosidase activity, downregulated the pro-oxidant enzyme NOX4, and upregulated SIRT1 and PTX3. These findings suggest that irisin, as part of this cocktail, contributes to modulating the NOX4–SIRT1 redox axis, thereby attenuating oxidative stress and cellular senescence and improving osteoblastic metabolism. Notably, the protective effects were more pronounced in osteoarthritic cells than in osteoporotic cells, indicating that irisin-mediated antioxidant actions may be influenced by the disease microenvironment and bone metabolic status. Overall, irisin may exert bone-protective effects by suppressing NOX4-derived ROS, activating SIRT1-related antioxidant signaling, and upregulating PTX3, although its independent contribution requires further validation (Bonanni et al., 2025).

### 4.9 Reproductive system

#### 4.9.1 Male reproduction

Oxidative stress adversely affects spermatogenesis, testicular steroidogenesis, and sperm quality. Excessive ROS damage sperm membrane lipids, impair mitochondrial function in spermatozoa, and increase DNA fragmentation. Shokrollahi et al. reported that exogenous irisin administration upregulates GnRH, LH, FSH, and testosterone secretion by activating the hypothalamic–pituitary–gonadal axis in male rats, while lowering ROS and MDA concentrations, boosting total antioxidant capacity, and increasing sperm counts (Shokrollahi et al., 2025). In obese male mice, irisin increases testicular steroidogenic enzyme expression, alleviates palmitate-induced endoplasmic reticulum and oxidative damage, reduces 4-HNE adduct formation, restores SOD activity, and preserves testicular function via Nrf2 activation (Mu et al., 2021). Irisin also protects Sertoli cell barrier integrity by regulating tight junction proteins under oxidative conditions (Lalruatmawii, 2026).

#### 4.9.2 Female reproduction and polycystic ovary syndrome

Hyperandrogenism in polycystic ovary syndrome (PCOS) triggers oxidative-stress-driven NLRP3 inflammasome activation and ferroptosis in ovarian granulosa cells, contributing to follicular arrest, anovulation and resultant follicular dysfunction. Exercise-induced irisin protects ovarian function by attenuating oxidative stress through complementary mechanisms. Weng et al. demonstrated that irisin inhibits the IRE1α–TXNIP/ROS–NLRP3 signalling pathway, thereby reducing ROS production, inflammation, and fibrosis in hyperandrogenic PCOS rats (Weng et al., 2023). Zhang et al. further showed that exercise-induced irisin suppresses ferroptosis by modulating the NCOA4-ferritin heavy chain (FTH) pathway, restoring iron homeostasis, reducing Fe^2+^ overload and oxidative stress, and preserving GPX4 expression in DHEA-induced PCOS mice (Zhang et al., 2026). Collectively, these findings indicate that irisin alleviates PCOS-associated ovarian injury by suppressing oxidative stress and ferroptosis via the IRE1α–TXNIP/ROS–NLRP3 and NCOA4–FTH pathways.

### 4.10 Aging and age-related diseases

Aging is characterized by progressive accumulation of oxidative damage, mitochondrial dysfunction, and cellular senescence, which collectively drive the development of age-related disorders including sarcopenia, cognitive decline, and metabolic dysfunction. Given that circulating irisin levels decline with age, strategies to restore irisin have attracted considerable interest (Huh et al., 2012; Colaianni et al., 2021). In the context of age-associated sarcopenia—defined by progressive loss of skeletal muscle mass and function—irisin has demonstrated promising therapeutic potential. Mao et al. reported that recombinant irisin treatment significantly increased serum irisin levels in aged mice and improved functional metrics including hanging time, grip strength, and muscle mass in a dosage-dependent manner. Mechanistically, irisin was found to modulate iron homeostasis and restore key oxidative stress-related proteins such as GPX4 and SLC7A11, potentially through the SIRT1/P53 pathway (Ma et al., 2025a). Ma et al. further demonstrated that Fndc5/irisin mediates the benefits of aerobic exercise on aging-associated sarcopenia in mice. A decline in Fndc5/irisin expression was detected in the gastrocnemius muscles with aging, and in Fndc5-knockout aged mice, aerobic exercise failed to improve skeletal muscle function, confirming the essential role of Fndc5/irisin in exercise-induced protection against sarcopenia (Ma et al., 2025b). In the brain, exercise-induced irisin crosses the blood–brain barrier and activates Nrf2-ARE signaling to enhance antioxidant capacity, reduce oxidative stress, and improve cognitive function in aged mice (Lee et al., 2025). These findings collectively position irisin as a promising therapeutic candidate for counteracting age-related degenerative processes across multiple organ systems (Table 2).

**TABLE 2:** Summary of organ-protective effects of irisin in systemic diseases.

Organ/System	Disease/Model	Major protective effects	Key mechanisms	References
Cardiovascular	Myocardial I/R	Improves LVEF; reduces infarct size; lowers troponin	SOD2 binding; PINK1/Parkin mitophagy; NOX2/4 inhibition; ER stress reduction	(Li et al., 2021a; Wang et al., 2018; Wang et al., 2017; Lu et al., 2020; Xiong et al., 2022)
​	Atherosclerosis	Reduces plaque burden; improves endothelial function	PKC-β/NOX inhibition; eNOS restoration; miR-126–5p upregulation	(Zhu et al., 2015; Zhang et al., 2016a; Zhang et al., 2016b; Wang et al., 2026a; Ismail et al., 2023)
​	Diabetic cardiomyopathy	Ameliorates diastolic dysfunction; reduces fibrosis	Integrin αVβ5-Akt; AMPK/mTOR autophagy; TGF-β1/Smad inhibition	(Lin et al., 2021; Deng et al., 2020; Chen et al., 2019)
Nervous	Cerebral I/R	Reduces infarct volume; improves neurological scores	SIRT3/SOD2; Nrf2 activation; PI3K/Akt/mTOR; DRP1 inhibition	(Cong et al., 2025; Liu et al., 2023; Peng et al., 2017; Jin et al., 2021)
​	Parkinson’s disease	Protects dopaminergic neurons; improves motor function	NLRP3 inhibition; SIRT1/HIF-1α/PGC-1α; autophagy enhancement	(Zhu et al., 2025; Cai et al., 2025)
​	Traumatic brain injury	Reduces neuronal apoptosis; preserves BBB integrity	SIRT3-dependent mitochondrial quality control; UCP2; AMPK/MerTK autophagy; SYK suppression	(Ge et al., 2024; Guo et al., 2021; Zhang et al., 2025b)
​	Prion disease	Rescues neuronal apoptosis	UCP2; AMPK–Nrf2	(Wen et al., 2025)
​	Sepsis-associated encephalopathy	Alleviates cognitive impairment; reduces neurological severity scores	Nrf2/GPX4 anti-ferroptosis	(Wang et al., 2022a)
Metabolic	Type 2 diabetes/β-cell	Preserves β-cell mass; improves insulin secretion	STAT3/Trx2; NLRP3/GSDMD; Nrf2–Trx/Txnip	(Liu et al., 2022b; Li et al., 2023; Liu et al., 2013)
Hepatic	NAFLD	Reduces steatosis and hepatocyte apoptosis	AMPKα; SREBP-1c; Nrf2/HO-1	(Canivet et al., 2020; Mertoğlu et al., 2025; Daneshi-Maskooni et al., 2019; Zhang et al., 2013; Polyzos et al., 2014; Yu et al., 2022)
​	Hepatic I/R	Reduces oxidative stress; inhibits ferroptosis	Integrin αV-dependent; DRP1 inhibition; MFN2/PGC-1α upregulation; GPX4	(Bi et al., 2019)
​	Liver fibrosis	Inhibits HSC activation; reduces fibrogenesis	TGF-β1/Smad inhibition; mitochondrial fusion-fission balance	(Dong et al., 2020; Lai et al., 2024)
​	Sepsis-associated liver injury	Suppresses ferroptosis; restores mitochondrial function	GPX4; NLRP3/NF-κB inhibition	(Wei et al., 2020; Li et al., 2021b)
Renal	Acute kidney injury (I/R)	Reduces tubular injury; restores renal function	UCP2/AMPK; GPX4; PGC-1α autophagy	(Zhang et al., 2020; Zhang et al., 2021; Han et al., 2026)
​	Sepsis-associated AKI	Preserves renal function; reduces histological damage	SIRT1/Nrf2; Nrf2/HO-1; AMPK/Nrf2 anti-ferroptosis	(Qiongyue et al., 2022; Wang et al., 2026b; Gui et al., 2024)
Respiratory	Acute lung injury	Reduces pulmonary damage; restores GSH	HSP90/NLRP3/caspase-1/GSDMD inhibition; AMPK/SIRT1; ferroptosis inhibition	(Zhang et al., 2025c; Han et al., 2023; Li et al., 2019)
​	Lung I/R	Protects alveolar-capillary barrier	UCP2; GPX4/ACSL4 balance	(Chen et al., 2017; Wang et al., 2022b)
​	COPD	Improves emphysema; reduces airway remodelling	Integrin αVβ5; PERK/ATF4/CHOP; AMPK; Nrf2/HO-1; TGF-β1 inhibition	(Ijiri et al., 2015; Sugiyama et al., 2017; Mandal et al., 2018; Bai et al., 2026; Kubo et al., 2019)
​	Asthma	Reduces airway fibrosis and inflammation	AMPK/Nrf2; Smad3 inhibition	(Bai et al., 2026)
Gastrointestinal	Intestinal I/R	Maintains epithelial barrier; reduces bacterial translocation	Integrin αVβ5-AMPK-UCP2; Nrf2 activation; NLRP3 inhibition	(Du et al., 2019; Bi et al., 2020; Fan et al., 2019)
​	Acute pancreatitis	Limits systemic inflammation; reduces acinar necrosis	KEAP1 degradation; mitochondrial ROS reduction; ER stress inhibition	(Tang et al., 2025; Ren et al., 2019; Ren et al., 2020)
Skeletal	Osteoporosis	Increases bone mass; promotes osteogenesis	SIRT3/SOD2; AMPK-PGC-1α; Nrf2; ROS-NF-κB inhibition	(Li et al., 2022; Colaianni et al., 2015; Hu et al., 2026)
​	Osteoarthritis	Inhibits chondrocyte ferroptosis; delays joint degeneration	NOX4 suppression; SIRT1 activation; PTX3 upregulation	(Bonanni et al., 2025)
Reproductive	Male fertility	Improves sperm quality; protects Sertoli cells	Nrf2; antioxidant enzyme restoration	(Shokrollahi et al., 2025; Mu et al., 2021; Lalruatmawii, 2026)
​	PCOS	Rescues follicular dysfunction; reduces ovarian ferroptosis	IRE1α-TXNIP/ROS-NLRP3; NCOA4-FTH	(Weng et al., 2023; Zhang et al., 2026)
Sepsis	Multi-organ dysfunction	Attenuates organ damage (liver, lung, kidney, heart)	Ferroptosis inhibition; mitochondrial protection; anti-inflammatory	(Wei et al., 2020; Qiongyue et al., 2022; Zhang et al., 2025c)
Aging	Sarcopenia; cognitive decline	Improves muscle mass and cognition	SIRT1/p53/GPX4; Nrf2-ARE; mitochondrial	(Ma et al., 2025a; Ma et al., 2025b; Lee et al., 2025)

## 5 Irisin as a potential biomarker of oxidative-stress-associated diseases

Serum irisin has attracted substantial interest as a non-invasive circulating biomarker. Reduced circulating irisin concentrations have been documented in patients with type 2 diabetes, coronary artery disease, heart failure, NAFLD, COPD, and multiple neurodegenerative disorders. In acute critical illnesses including sepsis and ARDS, low serum irisin correlates with greater disease severity. Nevertheless, several critical limitations hinder its clinical implementation as a standardized diagnostic biomarker. Commercially available enzyme-linked immunosorbent assay (ELISA) kits yield highly variable quantitative irisin measurements, ranging from ng/mL to μg/mL across different assays. Furthermore, circulating irisin levels are dynamically modulated by exercise intensity, exercise duration, and baseline metabolic status. Therefore, additional standardization work—including harmonization of antibody specificity, reference materials, and pre-analytical sample handling—is required before serum irisin can be adopted for routine clinical biomarker applications.

## 6 Therapeutic prospects and future research directions

### 6.1 Recombinant irisin and drug-development strategies

Recombinant human irisin has been successfully expressed in *Escherichia coli*, and purified recombinant protein retains biological activity in vitro functional assays (Panati et al., 2018). Systemic administration of recombinant irisin yields robust organ-protective antioxidant effects across diverse preclinical disease models. No overt adverse effects have been reported in these preclinical studies to date (Bi et al., 2019; Kam et al., 2022; Wu S. et al., 2023; Patel et al., 2026; Nguyen et al., 2024). However, the short half-life and potential immunogenicity of recombinant proteins pose challenges. Future development may focus on irisin mimetics, peptide analogues with enhanced stability, or small-molecule agonists targeting integrin αV receptors.

### 6.2 Physical exercise as a natural strategy to boost endogenous irisin production

Moderate-intensity aerobic exercise (65%–85% of maximal oxygen uptake) reliably elevates circulating irisin concentrations and activates tissue-protective downstream signalling (Ren et al., 2022). Exercise-induced irisin serves as a critical signalling mediator facilitating inter-organ crosstalk between skeletal muscle and distant target organs (Islam et al., 2021; Li H. et al., 2024; Shahabi et al., 2024; Wu et al., 2025). These mechanistic findings provide a physiological foundation for evidence-based exercise prescription in chronic oxidative-stress-related diseases. Personalized exercise regimens, tailored to individual cardiorespiratory fitness and disease status, may optimize endogenous irisin release and maximize therapeutic benefit.

### 6.3 Outstanding challenges and unresolved controversies for clinical translation

Several major obstacles remain to be overcome to translate irisin-targeted therapies from bench to bedside, including receptor heterogeneity, inter-species disparities, undefined pharmacokinetic profiles, context-dependent conflicting biological effects, and assay standardization. Specifically, while integrin αVβ1/αVβ5 are confirmed as irisin receptors, tissue-specific expression patterns and potential alternative receptor candidates have not been fully characterized, and understanding receptor distribution and downstream signalling specificity is crucial for predicting organ-selective effects; preclinical protective outcomes observed in rodent models are not always directly reproducible in human populations due to inter-species differences in irisin amino-acid sequence and metabolic clearance, warranting humanized animal models or non-human primate studies; the *in vivo* half-life, tissue-distribution characteristics, and optimal dosing regimens of exogenous recombinant irisin remain incompletely elucidated, necessitating detailed pharmacokinetic and pharmacodynamic studies to guide clinical trial design; several studies have reported divergent outcomes for irisin-mediated NF-κB regulation and cell-proliferation signalling under distinct cellular microenvironments, requiring further mechanistic clarification—particularly the potential dual role of irisin in tumour biology, which needs careful evaluation before systemic administration in cancer patients; and finally, the lack of a universally accepted reference method for irisin quantification hampers both biomarker validation and cross-study comparability, underscoring the essential need for collaborative efforts to establish international standards.

## 7 Conclusion

Irisin functions as a versatile endogenous antioxidant guardian, conferring widespread cytoprotection against oxidative-stress-induced injury throughout nearly all major organ systems. Its multifaceted antioxidant arsenal encompasses Nrf2/ARE transcriptional activation, SIRT3-driven mitochondrial SOD2 deacetylation, thioredoxin system modulation, mitochondrial dynamics regulation, and direct repression of pro-oxidant enzymes. Triggered by integrin receptor ligation, irisin orchestrates a coordinated cellular antioxidant response that simultaneously curtails ROS generation and amplifies endogenous ROS-scavenging capacity. When combined with its complementary anti-inflammatory, anti-apoptotic, and metabolic-regulatory activities, irisin emerges as a highly promising therapeutic candidate for oxidative-stress-related human diseases. Future translational research should prioritize the development of metabolically stable irisin analogues with improved pharmacokinetic properties, well-powered human clinical trials to validate therapeutic safety and efficacy, and in-depth characterization of tissue-specific irisin–receptor signalling networks, to maximize therapeutic benefit and minimize potential off-target adverse effects (Figure 2).

**FIGURE 2:**
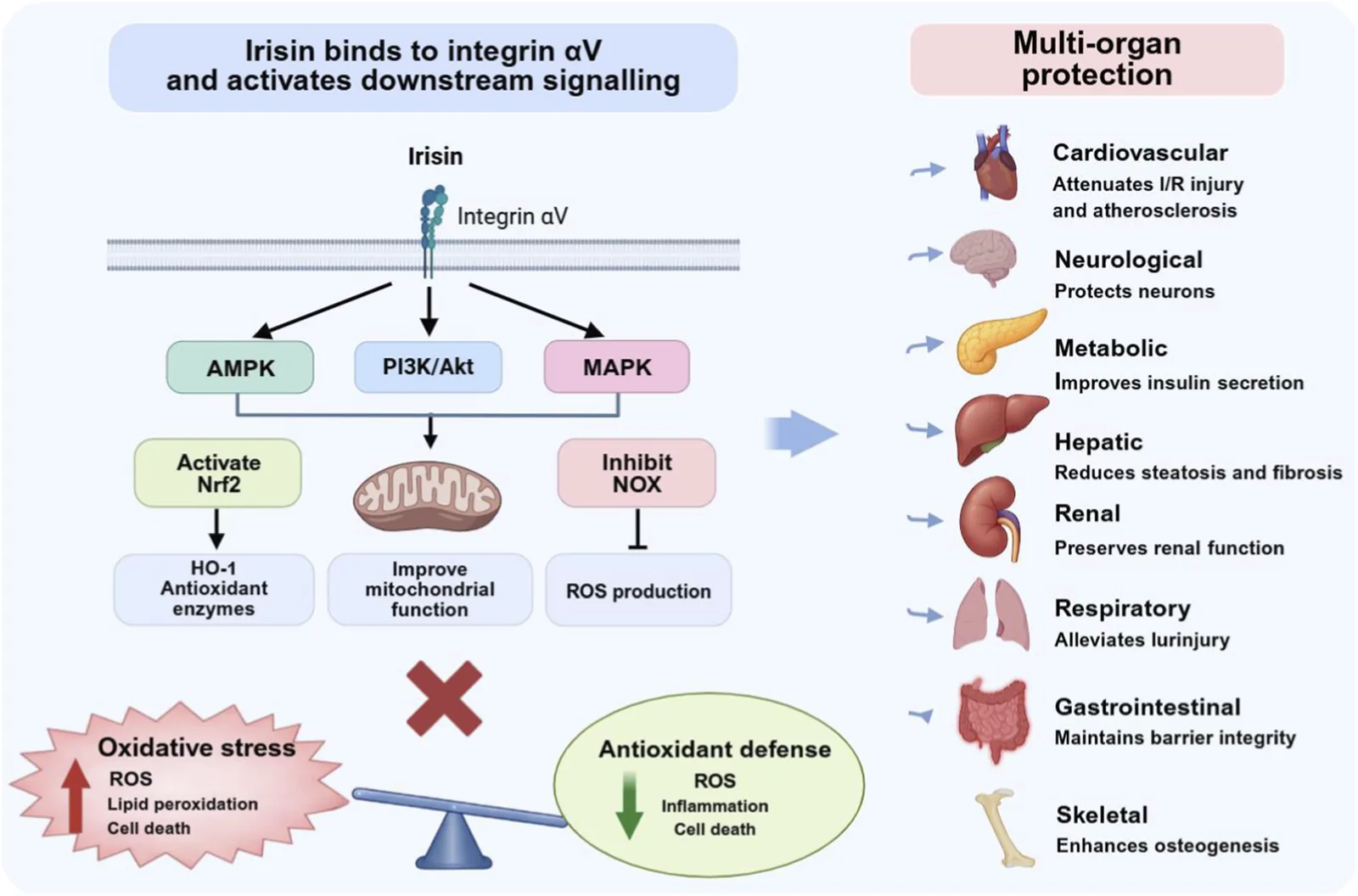
Molecular mechanisms and organ-protective effects of irisin against oxidative stress. Irisin protects against oxidative stress by activating Nrf2/ARE, SIRT3-SOD2, and STAT3-Trx2 signalling, regulating mitochondrial dynamics, and inhibiting pro-oxidant enzymes such as NOX, thereby conferring protection across the cardiovascular, nervous, metabolic, hepatic, renal, respiratory, gastrointestinal, skeletal, and reproductive systems.
